# A Stress Response Program at the Origin of Evolutionary Innovation in the Skin

**DOI:** 10.1177/1176934319862246

**Published:** 2019-07-03

**Authors:** Leopold Eckhart, Florian Ehrlich, Erwin Tschachler

**Affiliations:** Research Division of Biology and Pathobiology of the Skin, Department of Dermatology, Medical University of Vienna, Vienna, Austria

**Keywords:** Epidermis, evolution, stress response, keratin, cytoskeleton, cetaceans

## Abstract

The skin epithelium, ie, the epidermis, of dolphins and whales (cetaceans) is up to 50 times thicker than that of humans and other mammals living on land. Recently, comparative genomics revealed further striking differences in the cytoskeleton of the outer layers of the epidermis in aquatic and terrestrial mammals. Cetaceans lack the cytoskeletal keratins, which make up more than half of the total protein mass in the cornified epidermal layer of terrestrial mammals under homeostatic conditions. By contrast, orthologs of stress-inducible epithelial keratins are conserved in cetaceans and these keratins are constitutively expressed in their skin. Thus, the epidermal stress response program of a terrestrial common ancestor of modern mammals has become the default program of epidermal differentiation and a central component of the unique cutaneous organization of cetaceans. We propose that phenotypic plasticity during stress responses plays important roles in the evolution of the skin.

**Comment on:** Ehrlich F, Fischer H, Langbein L, et al. Differential evolution of the epidermal keratin cytoskeleton in terrestrial and aquatic mammals. *Mol Biol Evol*. 2019;36:328-340. doi: 10.1093/molbev/msy21. PMID: 30517738. PMCID: PMC6367960. https://www.ncbi.nlm.nih.gov/pmc/articles/PMC6367960/.

The skin is the body’s organ at the interface to the environment, and its main function is the protection against harmful insults from the outside and loss of water from the inside.^1^ The direct contact with the environment implies exposure to various kinds of stress within the lifetime of an organism and an important role of the skin during the evolution of species. While skin responses to external stress are major topics in dermatology and toxicology,^2^ the evolution of the terrestrial skin barrier against desiccation and the evolution of skin appendages such as hair and feathers are of great interest for evolutionary biology.^3^

We performed a comparative genomics study to determine differences in the cytoskeleton of the main epidermal cell type, the keratinocyte, of terrestrial and aquatic mammals, ie, cetaceans and sirenians. The cytoskeleton is crucial for resisting mechanical stress and constitutes around 75% to 85% of the total protein in cornified keratinocytes.^4,5^ The cytoskeleton of epidermal keratinocytes is formed by keratin intermediate filaments that consist of heterodimers of type I and type II keratin. An important feature of keratinocyte differentiation is the change of the cytoskeleton composition. In proliferating keratinocytes attached to the basement membrane of the epidermis, keratins K5 and K14 form the intermediate filaments, whereas in the keratinocytes that have stopped proliferation on movement to the suprabasal layers, K1 and K10 form the major part of the cytoskeleton. When the epidermis is wounded, keratinocytes that exit the basal layer do not switch to K1/K10 but rather to K6/K16 or K6/K17 expression.^6^ In addition, wound-healing epidermis gains in thickness relative to homeostatic epidermis because of increased keratinocyte proliferation. In addition, the activated keratinocytes contribute to a proinflammatory micromilieu by secreting cytokines that attract immune cells.

In humans and other mammals, K1/K10 and K6/16 are markers of 2 alternative keratinocyte differentiation programs, and the epidermis of all body sites can switch between these programs depending on conditions of homeostasis or tissue repair. Accordingly, both programs are conserved in phylogenetically diverse mammals.^7^ However, our study showed that there are 2 remarkable exceptions: cetaceans and sirenians. Both clades of fully aquatic mammals have lost the *Krt1* and *Krt10* genes. This is surprising and remarkable because K1 and K10 together represent more than half of the total protein in human superficial keratinocytes.^5^ How is the absence of these proteins compensated in keratinocytes of aquatic mammals? To answer this question, we investigated skin transcriptome data that were collected from dolphins^8^ and found high expression levels of K6 and K17 besides the basal layer keratins K5 and K14. Of note, K16 and K17 are similar in sequence and only K17 but not K16 is conserved in cetaceans and their closest terrestrial relatives (hippopotamus and ruminants). Our results indicate that the epidermal wound healing program of ancestral terrestrial mammals has become the constitutively active default epidermal differentiation program in whales and dolphins (Figure 1).^7^

**Figure 1.**
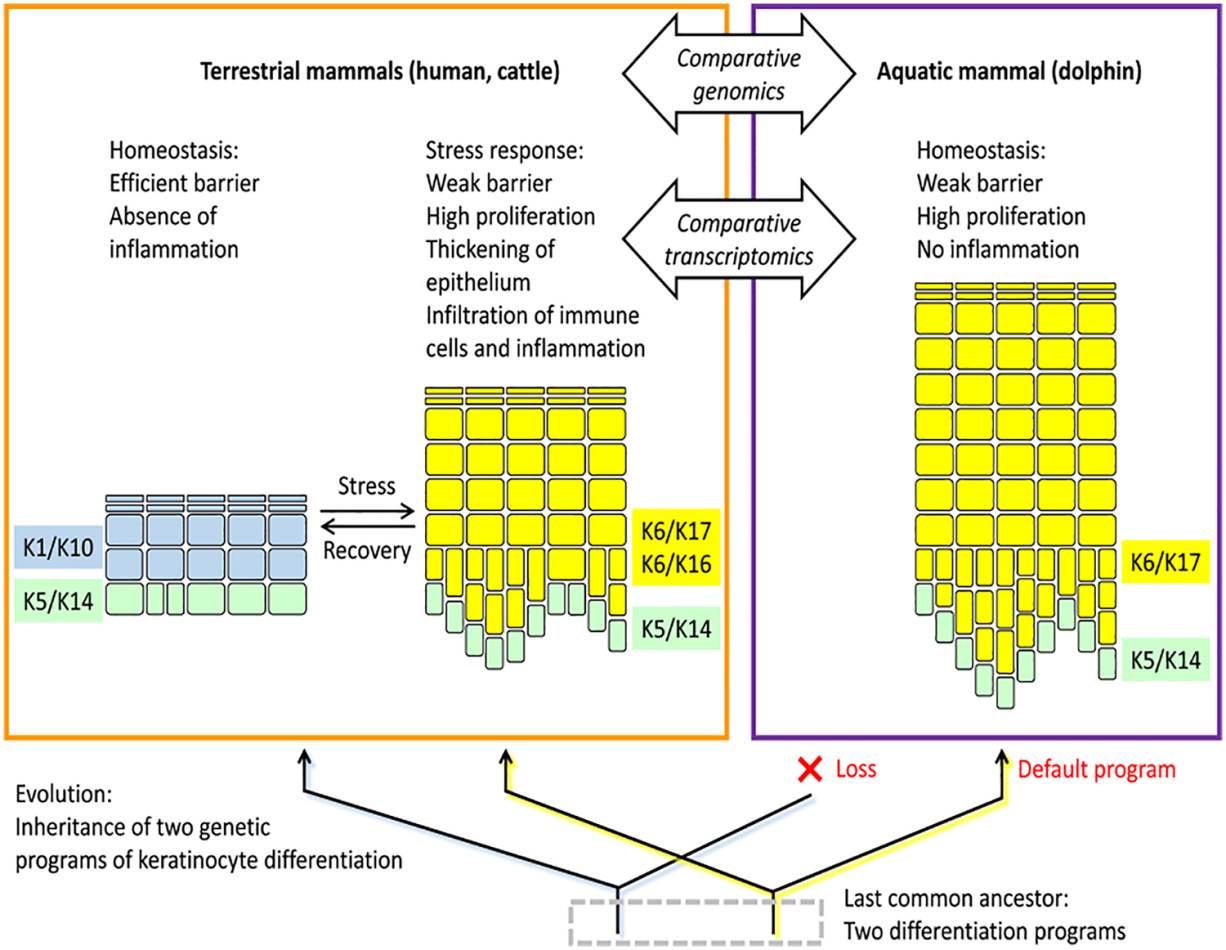
The epidermis of the dolphin evolved from a stress-inducible epidermal differentiation program of a terrestrial ancestor. The cellular organization of the epidermis in terrestrial and fully aquatic mammals is schematically depicted. The color of the cells (keratinocytes) indicates the composition of the keratin cytoskeleton. Keratins K5 and K14 are expressed in the epidermal basal layer where keratinocytes proliferate before undergoing terminal differentiation in the suprabasal layers. Comparative genomics and transcriptomics of terrestrial and aquatic mammals revealed that keratin markers (K6 and K17) of a stress program of terrestrial epidermis are constitutively expressed in the skin of dolphins, whereas the genes encoding the keratin markers (K1 and K10) of the homeostatic terrestrial skin barrier are lost in cetaceans. Comparative analyses of other important genes within the epidermal differentiation programs of terrestrial and aquatic mammals are likely to reveal insights into the control of mammalian skin barrier function, inflammation, and regeneration.

Although a wealth of gene expression data on human and murine epidermal keratinocytes under many conditions is available, there are few reports on the skin cells of cetaceans. In our study,^7^ gene expression in dolphin epidermis was inferred from transcriptome studies of dolphin skin, originally performed for monitoring environmental parameters.^8^ The determination of the epidermal keratin expression profile in a nonmodel species demonstrates that important insights into cell biology can be obtained if data sets from studies of different disciplines are combined.

As the ability to cope with stress has enabled the evolution of a new skin trait, ie, the extremely thick epidermis of cetaceans,^7^ it is tempting to speculate that the evolution of other skin structures also depended on stress-inducible differentiation programs. In particular, the evolution of the barrier against the nonaqueous environment in amphibians may have originated from a stress response program that was originally activated only on stress due to temporary drying-up of an aquatic habitate.

Our study^7^ focused on keratins, whereas a comprehensive investigation of epidermal transcriptome evolution in cetaceans is still missing. Keratins are the quantitatively predominant epidermal proteins, and they are used as markers of keratinocyte differentiation pathways in the clinic. However, the structure, dynamics, and metabolism undergo wide-ranging changes when the epidermis switches from homeostasis to regeneration and vice versa.^9^ Of note, many cellular processes are directly regulated by keratins.^10^ It will be exciting to determine which processes were deleted, maintained, or even expanded during the evolution of aquatic mammals. The adaptation of mammalian skin to fully aquatic life is just one of many evolutionary adaptations of the integument in mammals. For instance, diverse epidermal structures are present on mammalian toes and soles. Therefore, further studies are warranted to delineate which of the adapted epidermal differentiation programs evolved from stress programs.

The results of our study exemplify that phenotypic plasticity during stress responses can play an important role in evolutionary transitions of cell types and tissues. This concept is also supported by studies on the evolution of other biological systems, such as the early evolution of eyes^11^ and the evolution of pregnancy in placental mammals.^12,13^ We propose a key role of phenotypic plasticity due to genetic programs that are inducible in adult organisms. The ability to switch between genetic programs under different conditions allows the evolution of temporary and local skin features that differ dramatically from the homeostatic program and thereby allow changes in phenotypes that are not expected for gradual modifications of a single genetic program.
